# 1004. Case Study. Multimodal Plantar Reconstruction Using Dermal Substitutes, Hyperbaric Oxygen, and Negative Pressure Wound Therapy

**DOI:** 10.1093/jbcr/irag033.192

**Published:** 2026-04-06

**Authors:** Matthew Sanders, Mahmoud Hassouba, Dani Kruchevsky

**Affiliations:** Firefighters Regional One Burn Center, Memphis, TN; Firefighters Regional One Burn Center, Memphis, TN; Firefighters Regional One Burn Center, Memphis, TN

## Abstract

**Patient Presentation (age range, injury details, relevant history):**

A 45-year-old man with diabetes, severe neuropathy, chronic kidney disease, and prior kidney–pancreas transplant on immunosuppression presented with a 1.5% TBSA plantar full-thickness burn from prolonged heater contact. Exam showed leathery eschar, necrosis, absent PT pulse, and vascular/neuropathic skin changes.

**Clinical Challenges:**

Reconstruction was hindered by the plantar surface’s weight-bearing demands, exposure of plantar fascia, and severe comorbidities. Diabetes, CKD, neuropathy, immunosuppression, and PAD impaired healing capacity and perfusion. To optimize the wound be and promote favorable incorporation of dermal matrices and STSG, adjuncts like hyperbaric oxygen therapy (HBOT) and negative pressure wound therapy (NPWT) were employed to support salvage in this high-risk setting.

**Management Approach:**

A staged strategy combined excision, allograft, angioplasty, dermal substitutes, NPWT, and HBOT. Allograft conditioned the wound bed; angioplasty restored perfusion; Biodegradable Temporizing Matrix (BTM) and subsequently collagen-elastin wound matrix addressed exposed plantar fascia and bone. NPWT and HBOT enhanced dermal regeneration, enabling final STSG with stable take while minimizing morbidity and preserving function.

**Outcomes:**

Staged reconstruction with angioplasty, dermal matrices, NPWT, and STSG achieved durable closure. On areas that BTM didn’t incorporate favorably, estimated at 15% of the applied area, collagen-elastin wound matrix with NPWT was used to promote the development of optimized wound bed in a timely fashion. STSG demonstrated >90% take at 1 month with complete closure, maintained plantar contour, improved function, and avoided the need for a flap or amputation.

**Lessons Learned:**

Staged multimodal reconstruction was required in this complex plantar. Sequential use of allograft, BTM, collagen-elastin wound matrix, NPWT, and angioplasty enabled closure. The key takeaway from this case is that dermal substitutes are complementary, not interchangeable. It’s essential to be familiar with the strengths and limitations of each dermal substitute for a successful reconstruction.

**Applicability to Practice:**

This approach applies broadly to high-risk wounds in patient with severe comorbidities. Early vascular workup, combined dermal substitute use, and adjuncts such as NPWT/HBOT expand salvage options for patients with unfavorable prognosis.

